# 

**Published:** 1899-01

**Authors:** 


### INDEX TO VOLUME LIII.

PAGE.

COMMUNICATIONS.

Are Dentists a Set of Enthusiasts? ........................ 107

A Method of Handling Alveolar Pyorrhea................ 126

A Historical and Biographical Sketch of Geo. Watt, M.D., D.D.S. ..................209, 218

Bacteriology in the Treatment of Disease .................... 266

Cuspid Tooth in Relation to Character .................... 251

Concerning the Possibilities in the Art of Filling Teeth... 59

Civilized Mouth, The..... ...... 493

Dental Schools Abroad ......... 11

Dental Nomenclature............ 151

Dental Legislation............... 162

Dental Ethics ............. ...... 258

Detail in Cleft Palate Operations ......................... 501

Diseases of the Antrum of Highmore; A Study of 150 Cases...................... 459

Dental Education................. 409

Epithelial Structures in Peridental Membrane........... 374

Educational Methods........... 301

Filling Teeth...................... 124

Falsetto Voice in the Male..... 180

Foreign Relations Committee of the N. A. D. F........... 526

Infectious Ulcerative Stomatitis .............................. 360

Innervation of Tooth Pulp..... 51

Lecture with Stereopticon — Dental Caries, its Character, Cause and Effects...... 1

Notes on Pyorrhea Alveolaris. 17

New Treatment for Exposed Tooth Pulps................ 31

Operations for Cure of Chronic Empyema of the Antrum 101

Physiology and Pathology of the Pericementum......... 164

Periosteal Caries from Bacterial Origin................ 349

PAGE.

Points on the Etiology, Pathology and Treatment of Persistent Pyorrhea........ 368

Professional Ethics.............. 451

Pathology and Classification of Pulpless Teeth Without Regard to Abscessed Conditions........................ 397

Process of Refining and Manufacturing Gold Foil ...... 201

Sulphate of Magnesia........... 551

Syphilitic Infection................ 454

Therapeutics of Inflammation 405

The Human Face and Jaws as a Danger Signal of Systemic Defect or Disorder.. 410

The Relative Toxity of Cocain and Eucain..................... 445

The Maxillary Sinus—Chronic Empyema Thereof—Its Treatment .............. 186

Toothless Twentieth Centenarians, or Vegetarians—Which? ........................ 114

Tooth Forms in Relation to Jaw Movements............ 119

PROCEEDINGS.

A Simplified Fracture Splint.. 176

A Case in Oral Surgery ....... 177

Absorption Areas and Pulp Nodules........ ............... 274

A Remarkable Case ............. 272

A Statement to American Dentists Practicing in Europe 236

American Dental Society of Europe........................ 239

A Resume of the Important Changes that have Taken Place in Dentistry during the Last 35 Years .......... 512

Cements ... ......................... 544

Cement Question, Phases of.... 545

Conservative Antrum Treatment................................ 174

Chemistry, Metallurgy and Anatomy.......................... 227

PAGE.

Dental History .................... 469

Dental Articulation and Occlusion ........................ 470

Dental Caries, Susceptibility and Immunity to........... 547

Defective Tooth Development 228

Formaldehyde in Dentistry.... 275

Management of Children's Teeth ........................ 548

Microscopic Effects of Cataphoresis........................ 313

Microscopy, Histology and Bacteriology........ ......... 270

New Ohio State Dental Law... 36

Notes on Materia Medica, with Comments and Criticisms ........................225, 227

National Dental Association ........................463, 506, 544

National Association of Dental Faculties................... 412

Oral Hygiene........................ 314

Operative Dentistry.............. 223

Orthodontia and Oral Surgery 173

Ohio State Dental Society...... 129

Plain Rubber Teeth in Bridge Work ........................ 316

Porcelain Enamel Inlays....... 506

Resolutions.. ........ .............. 40

Report of Committee of Tri-State Meeting................ 129

Southern Branch, National Dental Association . ....... 313

Sectional Bridge Work.......... 315

Southern Branch, National Association of Dental Faculties ....... ............ 169

Tri-State Meeting.. .............. 71

The Advantages Offered in a Combination of Different Fillings ....................... 224

The Treatment of Pulpless Teeth by Iodoform Fumes Under Pressure.............. 227

The Dental Society of the State of New York... .............. 241

The Radical Cure of Cleft Palate ........................ 509

The Specialization of Our Preliminary Education......... 466

PAGE.

### SELECTIONS.

A Banquet without Wine...... 570

A Local and National Reproach ........................ 572

A New Anatomy ................. 573

A New York Oculist............ 58

Amalgams, The Shrinkage of 84

A Bacillus of Scurvy........... 88

A Mixture to Remove Freckles 95

A Registered Dentist Sued..... 96

Are We to be a Toothless Race? ........................ 135

Appropriation for the Johns Hopkins University........ 161

Are Athletes Healthy? ........ 194

An Unpleasant Experience with Cocain.................. 243

Abnormality of Hair Growth with Total Absence of Teeth ........................ 248

A Bill to Provide for Registration in N. South Wales 294

Ancient and Modern........... 325

A Note on a Case of Swallowing False Teeth............. 332

Anthropometry in Children... 429

A Decalogue of Hygienic Maxims ........................ 434

A Very Simple Procedure to Remove Moles...... ........ 434

Anesthetics........................ 435

A Bill Providing for the Organization of a State School of Public Health, Dr. N. J. Henry............ 438

A Movement to Perfect an Academy of Dental Science in San Francisco 437

A Certain Variety of Cold for which Gelsemium is as near Specific as any Drug can be........................ 439

A Movement has been Started 478

A Keen Diagnosis................ 480

An Enforced Anti-Spitting Law ........................ 485

A Source of Riches............... 486

A Supreme Court Decision..... 487

An Enviable Principality..... 517

Alcohol—Influence on Muscular Work.. ..................... 518

PAGE.

All Hypodermic Injections may be Rendered Less Painful...... 525

As Others See Us...... 479

Anesthesia, Local...... 481

Anesthesia, Observations in... 520

Anesthesia, Nitrous Oxide Gas and Oxygen...... 522

Antidotal Properties of Permanganate of Potassium.. 571

Bacilli in the Air and Infection...... 431

By the Will of the late Dr. Chas. J. Stille...... 479

Barbaric Dentistry...... 482

Bacteriologic Enzymes as the Cause of Acquired Immunity...... 484

Bacteriology...... 521

Buddha Tooth...... 435

Board of Health of the State of Rhode Island...... 483

Chloroform Syncope...... 80

Cocain, An Unpleasant Experience with...... 243

Carbolic Acid Dangerous Every Way...... 440

Changes in Salivary Glands in Diabetes...... 472

Contagion and Infection...... 477

Cold, To Abort a...... 436

Cause of Lamp Explosions...... 521

Cold Morning Bath, The...... 482

Chloroform, Death by...... 484

Cadavers for Anatomic Studies, Preservation of... 524

Dangers of Colds...... 569

Don't Forget Gen'l Wood...... 134

Dentist and Patient, Relations Between...... 241

Durability of Osteo Fillings... 329

Dental Board of Victoria, The 478

Death by Chloroforn...... 484

Dental Dispensaries...... 433

Decalogue of Hygienic Maxims, A...... 434

Dental Technics, National School of...... 579

Doctors and Health...... 522

Disinfection of Railway Coaches...... 525

Death, Unnatural...... 196

PAGE.

Disinfectant, The Best...... 248

Experiments with Nirvanin... 408

Eczema of the Lips Caused by Mouth Washes and Tooth Powders...... 481

Earache...... 481

Examining Michigan Recruits...... 482

Early Decay of Teeth in Britain...... 523

Effects of Mixed Diets on Salivary Digestion...... 87

Evolution of Decay...... 557

Foreign Body in the Throat... 92

Four Arguments Against Noise...... 93

Fahrenheit and Centigrade... 94

Foreign Body Imbedded in the Cheek for Eight Months.. 330

Far East, In the...... 408

"Forced" Education...... 524

French Therapeutics of the Period...... 322

False Teeth, A Note on Swallowing...... 332

Growth of Human Hair...... 486

Goethe as an Anatomist...... 518

Heredity...... 86

Hygienic Value of the Sun's Rays...... 195

Hints, Useful...... 296

Habit, a Factor to be Considered in the Treatment of Regulation Cases...... 437

High Altitudes and the Blood Count...... 475

Herr Metzger...... 433

Ignorance and Infant Mortality...... 144

Influence of the Quality of Drinking Water in the Structure of the Teeth.... 245

Influence of Sunlight upon the Human Body...... 295

Inquest on the Body of Harry Wesley Boyle Haines...... 334

Influence of Speech Disturbances on Psychical Development...... 430

Invention of Spectacles...... 483

Iodoformogene...... 486

Illinois State Board of Health 526

PAGE.

Jaborandi an Exceedingly Valuable Remedy.......... 197

Long-Delayed Amended Dental Bill....... ................ 96

Milk and Infantile Mortality 85

Meager Diet of Porto Rican Peasantry .................... 146

Medical College Consolidation 200

Mouth Breathing.................. 319

Modern Parisian Practice...... 322

Morphinism Among Physicians 575

Nitro Hydrochloric Acid in Dentistry.......................... 247

Nerve Transplantation......... 331

Never Sleep....... ................. 439

Nerve Tire in the Schools...... 515

Origin of Spectacles, The....... 91

Orthoform for Toothache...... 193

On Recent Methods of Treating Diseased Pulp.......... 246

Ocean and High Altitude Health Resorts.............. 519

Prevention of Disease............ 190

Plaster of Paris, Setting of...... 333

Plaster of Paris.................. 480

Physiology of Grafting......... 484

Pulp-Capping ..................... 487

Pure-Food Legislation.......... 567

Pure Drinking Water........... 520

Recovery of a Lost Nose........ 198

Relations Between Dentist and Patient ....................... 241

Report of Law Committee...... 575

Recent Methods of Treating Diseased Pulp... .......... 246

Remarkable how much Salt Water a Colon will Absorb .............................. 487

Sodium Salicylate for Tooth ache...................... ..... 146

Stuttering .............. ......... 248

Schemes for Hastening the Millennium .................. 477

Stomatitis Due to Milk......... 519

Standard Preliminary to a Medical Education......... 486

The Causation of Chloroform Syncope... .................... 80

Thymol as a Succedaneum for Arsenic in Pulpitis........ 94

Tape Worm.......... .......... 97

Ten Prize Hygienic Rules . ... 197

PAGE.

The Tongue in Influenza........ 432

The New Anesthetics........... 435

To Keep Needles and Cutting Instruments Bright... ..... 436

The Teeth of Recruits........... 439

The Immorality of Christian Science ...........................574

The Value of Concentrated Foods ........ ................... 473

Training the Hand............... 476

Temperance Restaurants........ 480

The Anthrax Bacillus Does Not Form Toxins........... 487

The Voice and the Circulation 523

Toothache.......... ............... 525

Toledo Dental Society .......... 579

Use and Abuse of Hypnotics in Insomnia................. . 82

Use of Formalin in the Treatment and Removal of Inoperable Malignant Growth. ...................... 438

University for Woman in Moscow.......... ................ 532

Vaccination and Beauty........ 90

What Hypodermatic Tablets Ought To Be................. 41

White Bread or Brown?........ 89

When We Were Young, Tom 440

When Russia Takes any Matter in Hand................. 483

COMMENCEMENTS.

Baltimore College of Dental Surgery ......................... 283

Baltimore Medical College, Dental Department......... 292

Birmingham Dental College... 293

Chicago College of Dental Surgery ............................. 281

Cincinnati College of Dental Surgery ........................ 286

Central College of Dentistry... 292

Colorado College of Dental Surgery ..... .................. 335

Columbian University, Washington, D.C., Dental Department ...................... 335

College of Physicians and Surgeons of San Francisco.... 338

PAGE.

Dental College of the Province of Quebec .......... 336

Illinois School of Dentistry .... 293

Indiana Dental College .......... 339

Kansas City Dental College ... 291

Keokuk Dental College ......... 340

Marion Simms College of Medicine, Dental Department 281

Milwaukee Medical College, Dental Department ......... 292

Medico-Chirurgical College of Philadelphia, Dental Department .......... 335

Missouri Dental College ........ 337

Miami Dental College .......... 340

Northwestern University Dental School .......... 280

New York Dental School ...... 334

Ohio College of Dental Surgery 336

Ohio Medical University, Department of Dentistry ...... 339

Pennsylvania College of Dental Surgery .......... 283

Philadelphia Dental College ... 284

Pittsburgh Dental College ...... 287

Southern Medical College, Dental Department ......... 288

University of California, College of Dentistry ............ 279

University of Maryland, Department of Dental Surgery .......... 285

University of Buffalo ........ 288, 290

University of Omaha, Dental Department ............ 289, 293

University of Tennessee, Dental Department ............ 290

University of Michigan, Dental Department .............. 337

Vanderbilt University, Dental Department .......... 289

Western Dental College ......... 291

Western Reserve University Dental College .............. 338

EDITORIAL.

American Dental Association .. 393

A Good Appliance .............. 346

At Last .......... 346

American Medical Association, Section on Stomatology .......... 299

PAGE.

An Important Movement ....... 395

Bibliographical .......... 199

Boston Dental College .......... 345

College Opening .......... 442

Care of Children's Teeth ........ 98

Commencements .......... 250

Dentists in the Army and Navy 45

Directory for Dental Societies and Colleges .......... 48

Examiners' Meeting ............ 395

Hygiene of the Mouth, a Guide to the Prevention and Control of Dental Caries ....... 347

Johnson's Universal Cylopedia 396

Look Out! Under $45,000 Bonds .......... 537

National Dental Association ... 441

National Association of Dental Faculties .......... 394

National Dental Association ... 298

Michigan State Dental Society 298

Ohio State Dental Society Annual Meeting .......... 492

Prosecution .......... 297

Pennsylvania State Dental Society .......... 492

Special Interest to the Dental Profession .......... 490

Regard for Law .......... 100

War Against Bogus Colleges and Degrees .......... 43

Who Shall Prepare our Food? 47

NOTICES.

American Medical Association 147

Annual Meeting of the National Association of Dental Faculties .......... 249

Board of Dental Examiners of Pennsylvania .......... 149

British Dental Journal ......... 150

Chicago Dental Society ......... 43

Examinations .......... 489

Fortieth Annual Meeting of the Northern Ohio Dental Association .......... 97

International Dental Congress, Paris, 1900 .......... 249, 532

Married .......... 149

Northern Ohio Dental Association .......... 198

PAGE.

National Dental Association Meeting ........................ 340
Second Annual Meeting of the Southern Branch of the National Dental Association ........................ 42
Sixteenth Annual Session of the National Association of Dental Examiners...... 296
Tennessee Dental Association.. 296
Thirty-fifth Annual Meeting Missouri State Dental Association........................ 297

PAGE.

Twenty-ninth Annual Session of the New Jersey State Dental Society........ ...... 345
Transportation Committee...... 533
Union Meeting Seventh and Eighth District Dental Societies, State of New York 488

BIOGRAPHICAL

Brophy, Emma J................ 150

Hamilton, Dr. J. B................ 49

Jones, Dr. Charles S.............. 444

St. John, Dr. I. C............ ...... 300

Wilson, Dr. F. D.................... 100

### NOTICES.

### Second Annual Meeting of the Southern Branch of the National Dental Association.

The Southern Branch of the National Dental Association, by invitation of the Louisiana State Dental Society, will hold its Second Annual Meeting in New Orleans, La., February 9, 10, 11 and 13, 1899, the following day being Mardi Gras. Circulars will be issued later giving railroad and hotel rates, etc. All members of the National Dental Association and the American Medical Association are cordially invited as guests of the Southern Branch.

WM. ERNEST WALKER,
*President Southern Branch, N. D. A.*

The following is a list of hotels and boarding-houses the visiting members of the Southern Branch of the National Dental Association may find convenient and desirable while attending the meeting. It will be necessary to notify the proprietor at least ten days in advance to secure good rooms:

HOTELS.—St. Charles Hotel, American plan, $4.00 per day and up, according to location of room. European plan, $2.50 and upwards. Hotel Royal, (French quarter) American or European plan, $2.50 to $4.00 per day, according to location of room. Cosmopolitan Hotel, European plan, from $1.50 upwards. Antoine's Hotel, (French) Nos. 713 to 717 St. Louis Street, $2.00 to $3.00. Hotel Denechaud, American or European plan, from $2.00 to $3.50 per day. Metrepole Hotel, American plan, $2.00 per day.

ROOMS.—Fabacher's Hotel, 50 cents to $1.00 per day. Penn's Hotel, 50 cents to $1.00 per day. Commercial Hotel, $1.00 to $2.00 per day. Metrepole Hotel, 50 cents to $1.00 per day. Rooms (French) Nos. 234, 237, 239 Bourban Street, from $1.00 to $3.00.

PRIVATE FAMILIES.—Mrs. Hawze, No. 1350 Magazine Street, rooms with board, $1.00 to $2.00 per day. Special rates by the week or month. Mrs. Joseph B. Davis, No. 1710 Prytania Street. Mrs. C. R. Van Wick, No. 1819 Annunciation Street. Miss Lulu Bailey, No. 846 Camp Street.

Any information concerning the above will be cheerfully furnished by the Chairman of the Hotels and Quarters Committee, Dr. J. Rollo Knopp, 620 Canal Street, New Orleans, La., or by Dr. Wallace Wood, Jr., 625 Canal Street, New Orleans, the Secretary of the Executive Committee of the Louisiana State Dental Society.

### Chicago Dental Society.

The Chicago Dental Society will, on Friday and Saturday, February 3d and 4th, celebrate its thirty-fifth anniversary by holding a two-day meeting for clinics, papers and discussions, ending with a banquet Saturday night. A cordial invitation is extended to the profession to be present. Printed programs will be ready in January, and may be had upon application.

JOSEPH W. WASSALL,
*Sec'y Committee of Arrangements.*

### EDITORIAL.

NOTICES.

Second Annual Meeting of the Southern Branch of the National Dental Association ........ 42
Chicago Dental Society ........ 43

EDITORIAL.

War Against Bogus Colleges and Degrees ........ 43

Dentists in the Army and Navy ........ 45

Who Shall Prepare Our Food? ........ 47

Directory for Dental Societies and Colleges ........ 48

OBITUARY.

Dr. J. B. Hamilton ........ 49

---

### THE DENTAL REGISTER,

**A MONTHLY MAGAZINE DEVOTED TO THE INTERESTS OF THE**

### DENTAL PROFESSION.

*Terms Reduced to* $2.00 *Per Year. Single Copies,* 25 *Cents.*

### EDITED BY PROF. J. TAFT, D.D.S.

—AND—

### Published by SAM'L A. CROCKER & CO., Ohio Dental and Surgical Depot.

The volume commences in January and closes in December.

The REGISTER being issued monthly is always fresh, therefore Indispensable to the practitioner who desires to keep posted up with the new inventions and improvements which are daily developed. Neither expense nor effort will be spared to give value and variety to its contents. Articles will be illustrated with well executed engravings whenever they will add to the interest or assist to explanation.

Its extensive circulation and frequency of issue make it one of the BEST DENTAL ADVERTISING MEDIUMS in the United States.

All subscriptions must begin with January or July.

Local or special notices, five lines or less, will be inserted for $3 each insertion; over five lines, 50 cts. per line.

All advertising bills payable in advance, or at furthest, after the issue of the first number containing the advertisement. All transient advertisements to be paid for in advance.

☞CONTRIBUTIONS SOLICITED.

Exclusively Editorial Communications should be addressed to the Editor.

The latest number of the REGISTER may be ordered with or without other good[illegible] any time, and all letters should be addres[illegible]

